# Three-port laparoscopic cholecystectomy in situs inversus totalis: A case report

**DOI:** 10.1097/MD.0000000000043186

**Published:** 2025-07-04

**Authors:** Hai-Tao Zhou, Jie Chen, Rui-Da Huang, Li-Meng Wang, Jian-Chun Zhou

**Affiliations:** a Department of Hepatobiliary & Pancreatic Surgery, Yuyao People’s Hospital, Ningbo, China.

**Keywords:** anatomical variations, laparoscopic cholecystectomy, situs inversus totalis

## Abstract

**Rationale:**

Situs inversus totalis (SIT), a congenital anomaly characterized by the mirror-image inversion of thoracic and abdominal viscera, necessitates a cautious approach in the diagnosis and treatment of patients presenting with symptomatic cholelithiasis. Laparoscopic cholecystectomy, the preferred procedure for gallbladder removal in SIT patients, achieves favorable outcomes through thorough preoperative planning, a deep understanding of anatomy, and intraoperative adaptability.

**Patient concerns:**

Herein, we present the case of a 51-year-old female patient in SIT who underwent a laparoscopic cholecystectomy without complications due to choledocholithiasis and gallbladder stones.

**Diagnosis:**

Gallbladder stone; SIT.

**Interventions:**

laparoscopic cholecystectomy.

**Outcomes:**

No complications such as bleeding or bile leakage (after LC) was detected. The patient was discharged after 2 days and recovered well after 1-year follow-up.

**Lessons:**

Thorough preoperative surgical planning, a deep understanding of anatomy, and the ability to adapt flexibly during surgery are key to the success of the operation.

## 1. Introduction

Situs inversus (SI) is a uncommon anatomical anomaly characterized by the transposition of internal organs to the opposite side of their normal anatomical positions.^[1]^ The prevalence of this condition ranges from 1 in 10,000 to 1 in 20,000 individuals. SI can be categorized into 2 types: situs inversus partialis (SIP) and situs inversus totalis (SIT). SIP primarily involves the transposition of thoracic organs (dextrocardia). In contrast, total situs inversus is a more rare variant, encompassing the reversal of both abdominal and thoracic viscera.^[2]^ The etiology of SIT remains poorly understood.

SIT is often associated with other congenital conditions, such as Kartagener syndrome (also known as primary ciliary dyskinesia), biliary atresia, and splenic malformations.^[3]^ In patients with SIT, the onset of cholecystitis presents unique challenges due to the altered anatomical locations of organs. The clinical manifestations of cholecystitis in these individuals may deviate from those observed in non-SIT individuals. The lack of experience among most surgeons in managing such cases can complicate the diagnostic and therapeutic processes, potentially leading to severe complications.^[4]^ Therefore, it is necessary to provide detailed reports on cases of cholelithiasis with SIT in order to increase the vigilance of surgeons.

## 2. Case presentation

Informed consent has been obtained from the patients and their families for the patient information involved in this case report. Our study was exempted from review, and this exemption complied with the policies of our institutional review board.

A 51-year-old female patient was admitted to the hepatobiliary surgery department with a primary complaint of left upper quadrant abdominal pain for 6 days. The pain was persistent and described as a bloating sensation, which extended to the left shoulder and back. The episodes were exacerbated during the night and following meals, accompanied by nausea without vomiting. Despite self-administering omeprazole orally during this period, the patient did not experience relief from the pain. Upon admission, the patient’s history and physical examination were conducted by the attending physician, revealing a previous diagnosis of depression. The patient is currently managed on a treatment regimen that includes paroxetine hydrochloride tablets, 20 mg daily, and olanzapine tablets, 5 mg daily.

**Figure 1.**
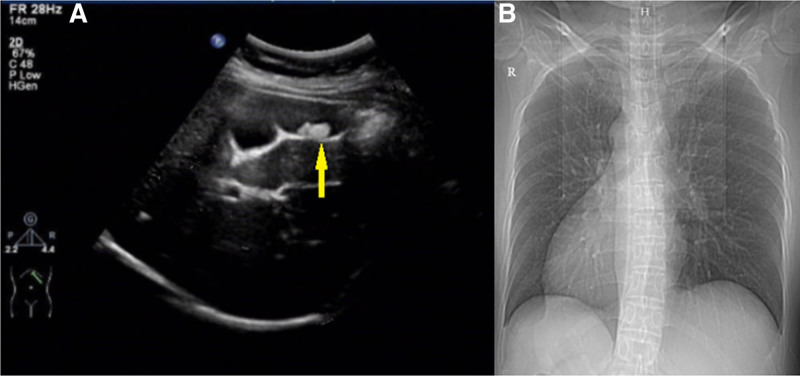
(A) Abdominal ultrasound image of the left upper quadrant showing a gallbladder with multiple stones (yellow arrow) (B) Chest X-ray demonstrated dextrocardia.

**Figure 2.**
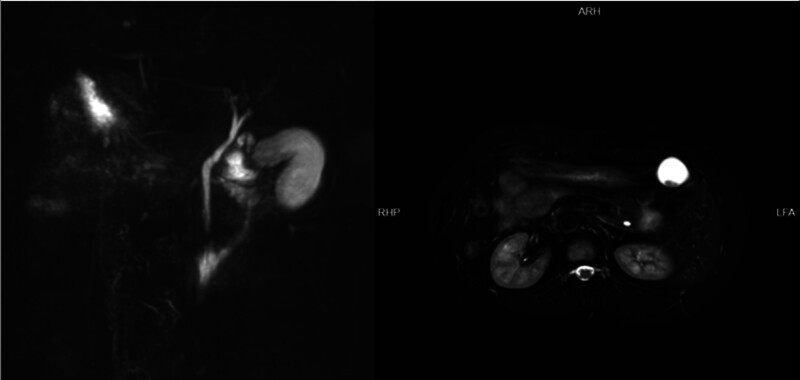
Magnetic resonance cholangiopancreatography (MRCP) scan confirmed the diagnosis of choledocholithiasis and gallbladder stones.

The patient has no history of previous abdominal surgeries, and there is no notable family history of medical conditions. Vital signs, including body temperature, pulse, and blood pressure, are within normal limits. The skin and sclera show no signs of jaundice. Cardiac auscultation detects a more intense right-sided heart sound without additional murmurs. Pulmonary examination reveals no abnormalities. The abdomen is soft to palpation, with tenderness localized to the left upper quadrant but without signs of rebound tenderness. The Murphy sign is negative. Chest radiography and abdominal ultrasonography revealed the coexistence of cholecystitis with gallstones, as well as SIT, as depicted in Figure 1. In alignment with the patient’s surgical preference, she was scheduled for hospitalization and an elective laparoscopic cholecystectomy (LC). Prior to surgery, an abdominal Magnetic Resonance Cholangiopancreatography (MRCP) was performed, with the findings detailed in Figure 2. Laboratory investigations were also conducted. The complete blood count, coagulation function, direct bilirubin, and total bilirubin levels were all within the normal range.

**Figure 3.**
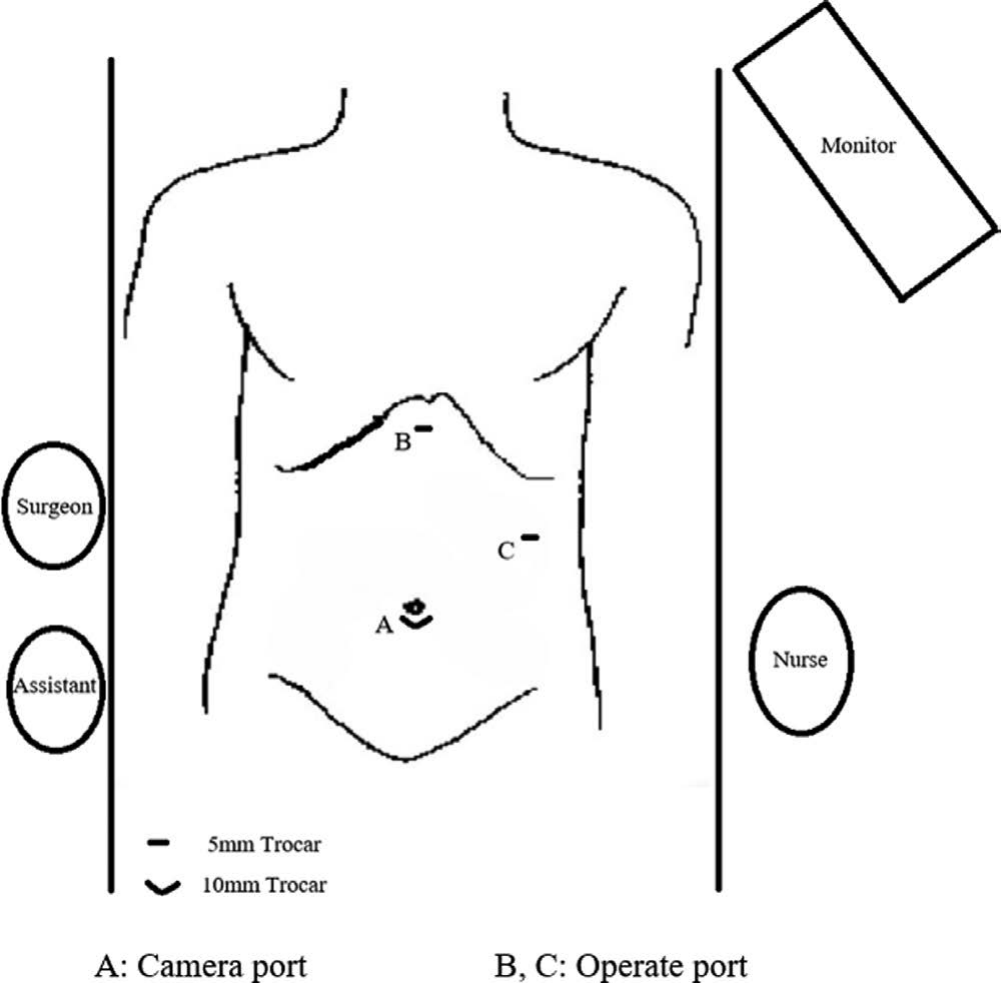
Adapted surgical positioning and trocar site placement for laparoscopic cholecystectomy in the patient with SIT. SIT = situs inversus totalis.

**Figure 4.**
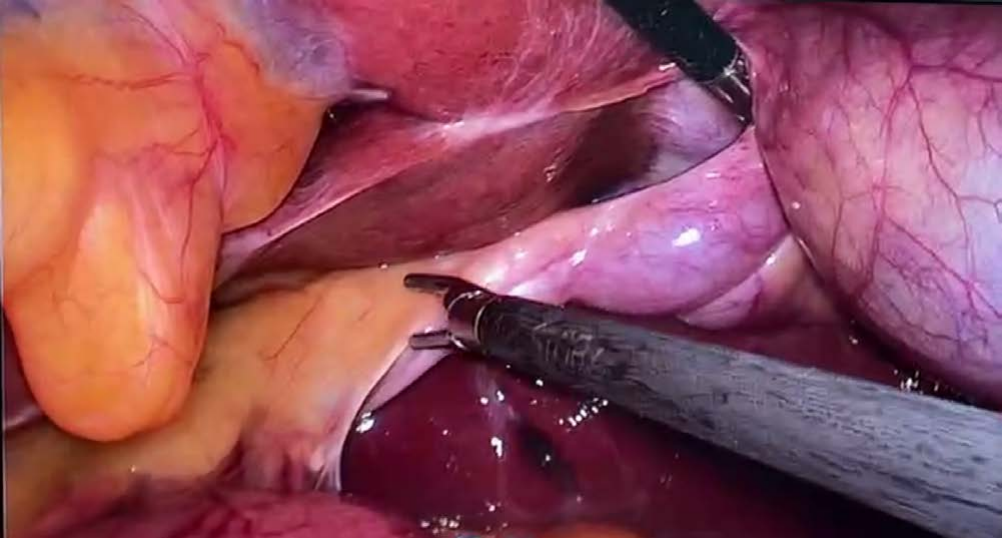
Laparoscopic image of intraoperative findings.

**Figure 5.**
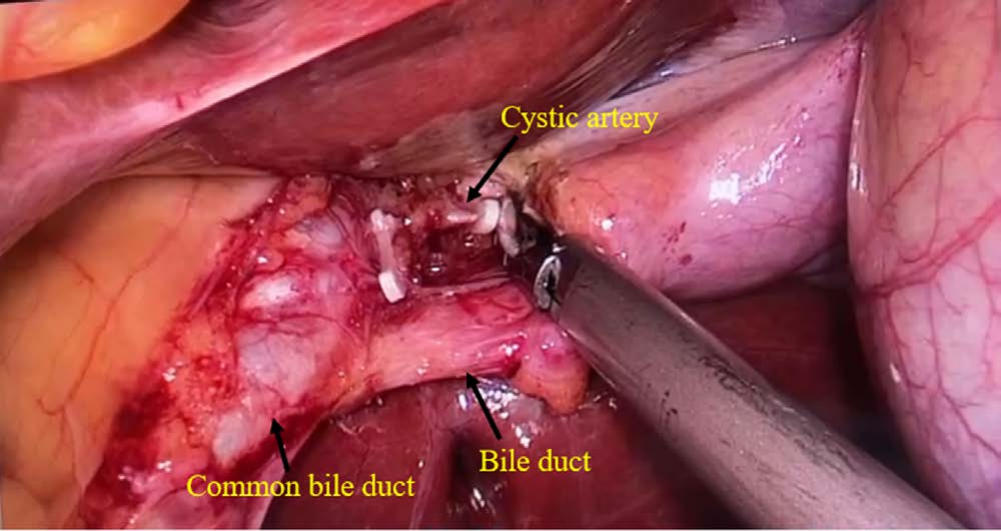
Intraoperative view of the cystic artery and bile duct.

The patient underwent a LC after excluding cardiopulmonary diseases and surgical contraindications. The placement of the laparoscopic equipment and the location of the trocar holes differed from those in the conventional LC, as illustrated in Figure 3. After induction of general anesthesia and standard surgical preparation, a 1-cm curved incision was made infra-umbilically. Carbon dioxide was insufflated into the abdominal cavity to achieve a pneumoperitoneum with a pressure of 14 mm Hg. Laparoscopic exploration was then conducted, which revealed a reversal of the positions of the abdominal organs, including the liver, spleen, stomach, and intestines, with the gallbladder situated in the left upper quadrant, as depicted in Figure 4. Taking into account the patient’s SIT, the patient’s position was adjusted to a supine head-up and feet-down tilt of 30° with a 15° rightward tilt. Both the primary surgeon and the assistant holding the laparoscope stood on the right side of the patient. Under direct laparoscopic visualization, 2 additional 5 mm Trocars were placed: 1 below the xiphoid process and another in the left upper abdomen. The surgeon’s left hand was used to lift the gallbladder’s fundus through the subxiphoid port, exposing the gallbladder triangle by pulling towards the left upper side. The right hand was used for dissection through the left upper abdominal port, traversing the anterior and posterior triangles of the gallbladder. Careful dissection revealed the cystic artery and bile duct, as shown in Figure 5. After repeated verification for accuracy, the structures were clipped with Hemolok and divided. The gallbladder is separated from its bed using an electrocautery hook. Once it is confirmed that there is no active hemorrhage or bile leakage, the gallbladder is extracted through the umbilical puncture site. Subsequently, all ports are sutured and dressed appropriately. Upon dissection of the gallbladder, a multitude of small calculi are identified, measuring between 0.3 cm and 0.5 cm in diameter. The operation time is 65 minutes. The final pathological report confirms the presence of gallstones and chronic cholecystitis.

The patient was discharged from the hospital 2 days following the surgical procedure, having made a satisfactory recovery. A 1-year outpatient follow-up period was conducted, during which the patient reported no discomfort or complications related to the surgery.

## 3. Discussion

SIT is a rare congenital anomaly characterized by autosomal recessive inheritance, which has been linked to gene mutations on chromosomes 7, 8, and 14. Clinically, it presents as a mirror-image reversal of abdominal and thoracic viscera.^[5]^ Although there is a significant alteration in the position of the organs, it does not affect their normal functional operation. Patients with total SI and cholecystolithiasis typically experience pain in the left upper abdomen, which clinically resembles acute pancreatitis and may even be misdiagnosed as gastritis. However, there are reports of 10% of patients presenting with typical right-sided abdominal pain, suggesting that the central nervous system’s processing of pain signals may not completely reverse according to the anatomical inversion.^[6]^ Therefore, routine physical examinations have certain limitations, which can make the diagnostic and treatment process confusing. For SIT patients requiring cholecystectomy, the unfamiliar abdominal anatomical inversion poses a challenging issue for surgeons. They must not only cautiously guard against variations in the bile ducts and blood vessels but also adapt to unconventional surgical practices, demonstrating exceptional adaptability during the operation.

In 1991, Campos and Sipes successfully performed the first cholecystectomy on a patient with SIT.^[7]^ Since then, similar case reports^[8–10]^ have emerged. documenting various surgical approaches, including the positioning of the surgeon and the placement of trocars. Enciu et al^[11]^ summarized 6 different surgical methods in a meta-analysis of 121 SIT patients undergoing LC. There have also been reports of successful surgeries performed with robotic assistance and the “single-port” approach.^[12,13]^ However, due to the rarity of SIT patients, large-scale comparative studies are not feasible, making it impossible to prove the superiority of any 1 surgical technique. We hope that sharing this case will help more young surgeons have adequate knowledge and preparation when facing similar cases.

Upon reviewing the literature, LC remains the procedure of choice for SIT patients requiring gallbladder removal, and it generally achieves favorable outcomes. However, there are still reports of intraoperative bile duct injuries occurring.^[4,14]^ Therefore, we believe that even experienced surgeons should not be complacent when facing similar cases, and currently, no scholars have proposed additional measures to reduce the risk of bile duct injury in these patients. Particularly in emergency surgery situations, surgeons often do not have sufficient preparation to deal with such anatomical variations, which undoubtedly increases the uncertainty of the operation and dramatically raises the risk of bile duct injury, further testing the surgeon’s adaptability.

Based on our experience, achieving the critical view of safety before dividing the cystic duct and repeatedly confirming the integrity of the biliary structures remains essential for the smooth completion of the surgery. Performing the surgery in the manner the surgeon is most proficient in is undoubtedly an important prerequisite for achieving the best treatment outcomes for the patient. Certainly, adjusting the patient’s position and the location of the abdominal trocars flexibly to increase the surgeon’s comfort during the operation can reduce the difficulty of intraoperative manipulation.

In this case, the change in the patient’s position greatly exposed the surgical field. Considering that the surgeon is right-handed, the left hand was used to lift the gallbladder fundus through the subxiphoid trocar to expose the Calot triangle, while the right hand performed the dissection through the left upper abdominal trocar. This approach maximized the surgeon’s comfort, promoting the completion of the surgery with high quality. Ultimately, we found that the operative duration was comparable to that of a standard LC (30–95 min) . This outcome can likely be attributed to the surgeon’s thorough preoperative preparation, including detailed case analysis and repeated mental rehearsal of the surgical steps.

Given that the surgeon is right-handed, dissecting Calot triangle with the left hand would inevitably increase both surgical risk and operator fatigue. Therefore, optimal trocar placement is critical. In our approach, a 12-mm infraumbilical port serves as the camera port, while the primary operating port for the right hand is placed in the left upper quadrant (2 cm above the umbilicus at the left midclavicular line). This configuration maximizes the right hand’s dexterity for Calot triangle dissection. Simultaneously, the left hand operates through a subxiphoid port to retract the gallbladder infundibulum, optimally exposing the medial and lateral borders of Calot triangle. This setup follows the “baseball diamond principle” of laparoscopic ergonomics, minimizing instrument readjustments, improving procedural precision and efficiency, and reducing operator fatigue.

It is necessary to conduct a thorough preoperative screening for SIT patients to exclude the presence of severe cardiopulmonary diseases. Reports indicate that the incidence of congenital heart disease in SIT patients ranges from 3% to 9%, which is significantly higher than that in the non-SIT population (0.6%).^[1]^ Fortunately, the case we report did not reveal any other congenital diseases. Comprehensive imaging studies such as preoperative MRCP, contrast-enhanced computed tomography (CT), and even endoscopic retrograde cholangiopancreatography (ERCP) are crucial for understanding biliary and vascular anomalies and avoiding postoperative complications. Only meticulous preoperative planning, thorough imaging examinations, and careful intraoperative dissection can ensure a smooth recovery for the patient. A key limitation of our study is the failure to implement 3-dimensional reconstruction from CT or MRCP imaging for preoperative planning. The application of this technology in future analogous cases would likely offer significant advantages to surgical practitioners.

## 4. Conclusions

SIT, a congenital anomaly characterized by the mirror-image inversion of thoracic and abdominal viscera, necessitates a cautious approach in the diagnosis and treatment of patients presenting with symptomatic cholelithiasis. Previous studies and clinical experience suggest that LC is the preferred modality for managing this condition. Thorough preoperative surgical planning, a deep understanding of anatomy, and the ability to adapt flexibly during surgery are key to the success of the operation.

## Author contributions

**Data curation:** Rui-Da Huang.

**Supervision:** Li-Meng Wang.

**Writing – original draft:** Hai-Tao Zhou.

**Writing – review & editing:** Jie Chen, Jian-Chun Zhou
